# Responsible innovation in synthetic biology in response to COVID-19: the role of data positionality

**DOI:** 10.1007/s10676-020-09565-9

**Published:** 2020-10-26

**Authors:** Koen Bruynseels

**Affiliations:** Philosophy Department, Technology Policy & Management, T.U. Delft, Jaffalaan 5, 2628 BX Delft, The Netherlands

**Keywords:** Positionality, Synthetic biology, Responsible innovation, Data frictions, Knowledge commons, Pandemic, COVID-19

## Abstract

Synthetic biology, as an engineering approach to biological systems, has the potential to disruptively innovate the development of vaccines, therapeutics, and diagnostics. Data accessibility and differences in data-usage capabilities are important factors in shaping this innovation landscape. In this paper, the data that underpin synthetic biology responses to the COVID-19 pandemic are analyzed as positional information goods—goods whose value depends on exclusivity. The positionality of biological data impacts the ability to guide innovations toward societally preferred goals. From both an ethical and economic point of view, positionality can lead to suboptimal as well as beneficial situations. When aiming for responsible innovation (i.e. embedding societal deliberation in the innovation process), it is important to consider hurdles and facilitators in data access and use. Central governance and knowledge commons provide routes to mitigate the negative effects of data positionality.

## Introduction

Synthetic biology is a bio-engineering field that pursues the data-driven design of biological systems (Freemont 2019). It combines molecular biology and lab automation with in silico design techniques that are fueled by biological data. In silico design refers to the computer-aided design of biological molecules and biological processes, for example, the modeling of proteins or the modeling of pathways that allow for the biochemical synthesis of compounds. Synthetic biology was highlighted in a report from the European Parliament as one of the emerging technologies that can fight the COVID-19 pandemic (Kritikos 2020). The National Institute of Health in the USA also identified synthetic biology as one way to speed up vaccine development (Begley 2020). Its potential to revolutionize the development and production of vaccines, therapeutics, and diagnostics underpins this hope. The techniques developed in the synthetic biology community open up radical new possibilities and allow for a more rapid exploration of such possibilities than with established processes. Synthetic biology labs and firms actively started applying their technologies to contribute solutions for the COVID-19 pandemic. Although probably not part of the first wave of drugs and vaccines, such innovations can shape future responses to this and all future pandemics. For example, DNA- and mRNA-based vaccine technologies can ease the development and production of vaccines. These vaccines consist of synthetic nucleotide strands that trigger the formation of proteins via the individual’s own cells, thereby inducing an immune response. The availability of viral sequence data can thus be rapidly translated into vaccine candidates. This allowed ventures such as Moderna and Inovio to move into clinical development in just a few months following the public release of the genetic code of the virus (Thanh Le et al. 2020). Synthetic biology techniques are also used to construct antigen-carrying nanoparticles. Such nanoparticles have been shown to effectively trigger immune responses in mice and nonhuman primates (Marcandalli et al. 2019). Nanoparticles can potentially reduce the need for adjuvants and facilitate scalable production. They also show high stability at room temperature, which would ease their distribution in low-income countries (Shin et al. 2020). This evolution testifies to the disruptive potential of data-driven “plug and play” platforms that aim at the modular design of vaccines against new viruses (CEPI 2020). Synthetic biology techniques have also been applied in drug discovery and development. For example, cell-free systems were used to design biosynthetic pathways for the antiviral agent valinomycin (Zhuang et al. 2020). Cell-free systems are free from the complexity and constraints that come with intact cells, containing only the biological components that support the process of interest. Such systems therefore have the potential to further widen the range of engineering possibilities. Synthetic biology techniques have also been applied to develop diagnostic tests for SARS-CoV-2 (Broughton et al. 2020).

The previous examples are indicative of the potential of synthetic biology techniques to disruptively transform how society can respond to viral outbreaks. Given the devastating impact of the COVID-19 pandemic on people, societies, and economies, rapid responses based on innovations in vaccine development, therapeutics, and diagnostics can be very beneficial. Innovations, however, need to be aligned with societal values to realize this potential. Besides biosafety and biosecurity, innovations need to align with values, such as privacy, access to good healthcare, and a fair distribution of derived benefits. Guiding innovation toward such societally preferred goals is highly relevant in view of the deluge of innovations in synthetic biology and the strong moral load of data-driven innovations in healthcare (Bruynseels et al. 2018). Responsible research and innovation (RRI) was proposed as a way to align technological innovation with values preferred by society. RRI has been explored in both synthetic biology (Macnaghten et al. 2016) and healthcare settings (Silva et al. 2018; Douglas and Stemerding 2013). By including social and ethical aspects in the innovation process, RRI provides a concrete approach for a moral accompaniment of technoscientific developments. RRI has been defined as “a transparent, interactive process by which societal actors and innovators become mutually responsive to each other with a view to the (ethical) acceptability, sustainability and societal desirability of the innovation process and its marketable products (in order to allow a proper embedding of scientific and technological advances in our society)” (Von Schomberg 2011) or as “taking care of the future through collective stewardship of science and innovation in the present” (Stilgoe et al. 2013). How, then, can the collective stewardship of innovations be organized in the case of synthetic biology?

Access to data is pivotal when pursuing synthetic biology innovations and is therefore important when pursuing RRI. As a bio-engineering practice, synthetic biology requires a close intertwinement of in silico discovery and modeling and automated lab experiments (Freemont 2019). Without access to genomic sequence data, high-quality sequence annotations, metabolic models, and so on, it is not possible to achieve much. Capabilities are also required to enable the data to be put to use: computational power, cutting-edge algorithms, and access to know-how (Sachsenmeier 2016). For COVID-19, excellent public resources are available. Full viral genome sequence data were published in the Global Initiative on Sharing All Influenza Data (GISAID 2020) and in Genbank’s SARS-CoV-2 data hub (GenBank SARS-CoV-2 2020) starting in early January 2020 (Holmes 2020; NHC 2020). Researchers swiftly used this information, for instance, to synthesize substitutes of the actual viral genome, thereby speeding up global research. Currently, hundreds of variants are available from locations across the globe. The COVID-19 Genomics UK Consortium (COG-UK) aims at sequencing SARS-CoV-2 viruses from up to 230,000 UK COVID-19 patients, with an underpinning commitment to open science and FAIR data principles [COVID-19 Genomics UK (COG-UK) consortium 2020].

Access to data can be a prerequisite for innovation. On the other hand, data frictions can hamper this ability to innovate. Data frictions (Edwards 2010; Bates 2018) are defined as “socio-material factors that coalesce to slow down and restrict data generation, movement and use.” Data frictions have a “politics”; they influence what is known by whom and therefore how future knowledge and social relations are shaped (Bates 2018). They are an important factor in shaping innovation because they impact which parties are involved. Data frictions relate to the kinds of data and the repositories they reside in, data standards, data-transfer mechanisms and policies, or the lack thereof. They comprise technical and societal hurdles that impair data access, as well as being catalysts that foster data access. During the outbreak of avian influenza A virus (H5N1), a stop placed on data sharing led to significant controversy. Indonesia stopped sharing clinical specimens to international laboratories participating in the World Health Organization Global Influenza Surveillance Network (Sedyaningsih et al. 2008). The rationale behind this was that sharing materials enables international companies to develop vaccines, but that the Indonesian population would not benefit from these developments. The demand for access to drugs and vaccines, for agreements on intellectual property rights, and for capabilities built up via technology transfer and scientific collaborations resulted in an international agreement on a “pandemic influenza preparedness framework.” Along the same lines, the MERS coronavirus was isolated in Saudi Arabia, but intellectual property rights on products based on the MERS genomic sequence were owned by a Dutch institute. This situation led to a significant dispute and questions about data sharing (Butler 2013). Data sharing was also hampered during the Ebola 2013–2015 outbreak due to a variety of hurdles (GRCIDP 2018). In some cases, viral genomic sequence data were swiftly uploaded via the public platform GenBank, but no standard method existed to disseminate the data. Most of the samples provided for genetic sequencing never resulted in publicly released data (Yozwiak et al. 2015). International initiatives recognized the need for improved data sharing, resulting in initiatives such as the establishment of GISAID, a platform for sharing influenza virus sequences and related epidemiological data (Bogner et al. 2006). These cases testify to the fact that it is crucial to organize access to data in such a way that innovation toward societally preferred goals is stimulated while risks are mitigated.

The data-driven innovation response to the COVID-19 pandemic exemplifies this point. Data positionality is put forward as a useful lens through which to analyze innovation dynamics in relation to data. Data positionality refers to situations where the value of data depends on the extent that others do not have access to that data. Positional goods’ theory was developed to describe a category of certain marketable goods whose value depends on externalities; namely, on how they compare with things owned by others (Hirsch 1976; Frank 1985; Pagano 1999; Vatiero 2009; Zinnbauer 2018). Positionality implies exclusivity: scarcity needs to be guaranteed and parties need to be able to benefit from the resulting exclusivity. The question that arises is how data positionality can impact the capability to steer innovation in synthetic biology in societally preferred directions. Positionality is related to the concept of data frictions (Edwards 2010; Bates 2018). Hurdles in accessing biological data determine whether and how data-driven innovation can be steered. Data access thus needs to be considered when aiming at responsible innovation. In this paper, the role of data in COVID-19-related innovations in synthetic biology is used to illustrate data positionality and its repercussions for responsible innovation.

## Synthetic biology data as a positional good

The rush for innovations in the wake of the COVID-19 pandemic is driven by both the pursuit of societal benefits and economic rationales. The current pursuit of drastically shortening vaccine development timelines (Thanh Le et al. 2020) testifies to this. COVID-19 vaccine development is embedded in a significant economic reality, where high investments are required to bring a vaccine to the market (Gouglas et al. 2018). CEPI is an organization that invests in vaccine development programs; part of its funding goes to synthetic biology companies (CEPI 2020).

Given this context, it is insightful to analyze the biological data used in synthetic biology in terms of *information goods* that have a market value. “Information goods” refers to commodities whose market value is determined by their information content and not by their material properties. Engineering approaches in synthetic biology can depend on a variety of information goods (genomic sequences, sequence annotations, enzyme properties, metabolic models, etc.), often from a variety of species. These goods also depend on lab protocols, algorithms, scientific knowledge, and technical know-how. The information goods that fuel innovations in synthetic biology are therefore very heterogeneous. In terms of the response to COVID-19, viral genomic sequence data and annotations, human genomic sequence data, and clinical and epidemiological data are all crucial inputs for innovations in prevention, diagnosis, and therapy.

What type of goods are information goods? Economic theory has various ways of classifying goods. Commonly, goods are categorized along two axes: according to their excludability and rivalry. Goods are excludable if parties can be denied access to them. Goods are subtractable (or rivalrous) if consumption by one party reduces the possible consumption by another party. Biological data are non-subtractable goods since consumption of the data by one party will not make the data unavailable to other parties. However, their production requires subtractable goods, such as time, money, and biological systems (e.g. ecosystems or populations). And the biological data themselves can result in subtractable goods (Strandburg et al. 2017), such as new medical treatments or washing powder enzymes. Biological information goods have often been categorized as public goods, which are non-excludable and non-subtractable. The rationale for this categorization is that they are a form of scientific knowledge, which is the archetypical example of a global public good. Scientific theories, such as Einstein’s relativity theory, are available to all, and usage of the theories does not diminish their value for others. This typecasting as a global public good can be used as a strategy to instill ethically preferred dynamics by stimulating the sharing of data across national and international boundaries (Chadwick and Wilson 2004). Moreover, in principle, well-oiled online markets that allow for efficient price-setting should result in information goods that cost virtually nothing. Digital artifacts can be copied at high speed and low cost as soon as the first artifact is made (Quah 2003). The synthetic biology community has tended to promote an ethos of open innovation (Torrance 2017), which is, at first sight, in conflict with the positional character of synthetic biology data. Along those lines, open-source software development is often used as inspiration when shaping the field (Urquiza-Garcia et al. 2019). The open-source software movement proved to be a very viable complement to proprietary software schemes, and it vastly stimulated innovation (Boyle 2008). Translated to the field of synthetic biology, this finds its analogy in schemes that allow for building freely on the genetically encoded functions shared by the community, such as through the BioBricks Public Agreement™ [BioBricks Public Agreement^(^™^)^ 2020]. Similarly, the open-science movement provides a model where scientific findings and related datasets are made publicly available without the hurdle of subscription costs (Levin et al. 2016; Burgelman et al. 2019). The synthetic biology community’s response to the corona pandemic showed that publicly available information can vastly speed up innovation.

In practice, the categories of biological information goods are much more colorful. Rather than being a public good, many datasets are not publicly available but reside in proprietary databases, experience delays or incompleteness in data release, or are only accessible given the right membership. Intellectual property regimes further complicate the picture by providing temporal monopolies over the concrete applications of patented knowledge. The ambiguity of synthetic biology in this respect is indicative. Synthetic biology is often defined as an engineering discipline, next to it being a scientific discipline. This hints at the fact that synthetic biology does not only result in scientific theories that are asymptotically available to all. In many cases, the outputs concern designs and engineered systems that provide a competitive advantage in a market, and thus are inherently related to information asymmetries. In economic terms, these observations are indicative of a market failure that leads to a tendency for some information to become exclusive rather than being free and open (Zinnbauer 2018). The value of some information goods depends on whether others do not own them; thus, on their exclusivity. Having priority access to biological data puts one in the position to mine the data first and produce derived goods, such as scientific papers, new pharmaceuticals, medical treatments, etc. Such information goods are more valuable if others do not have equal access to them or are less capable of putting these data to use. Digital information goods therefore do not, by definition, result in open data or in a market-clearing price that is close to zero. They can experience scarcities that are either artificially constructed or are the result of socio-technical constraints in data movement.

## Drives behind positional effects in biological information goods

Positionality has recently been described as an overlooked property of information goods in general, which can explain certain failures in the data market (Zinnbauer 2018). Instead of everyone enjoying a world of free and open data, many data-holders benefit from constructing an artificial scarcity in information so that a much higher premium can be gained. And there are buyers who are willing to pay these high premiums as long as the scarcity remains guaranteed and they can benefit from exclusivity. This holds true for premium political, business, and legal information, and also for certain forms of scientific information (Zinnbauer 2018). Many biological data and much derived knowledge reside in databases with tightly controlled access—often proprietary—and they are sometimes subject to intellectual property rights or expensive subscriptions. Thus, the introduction of a “manufactured scarcity” (Zinnbauer 2018) counteracts the fact that the data themselves are, in principle, infinitely sharable. In economic terms, such effects are called “positional.” Positional goods (Hirsch 1976) have a value that is determined in relative terms by their externalities. Their value does not merely depend on the quantity of the goods, but on their exclusivity—on the extent to which others have no access to them.

The positionality of information goods comes in various forms. At an abstract level, one can distinguish between horizontal and vertical positionality, depending on the types of externalities that impact the value of the information good. Vertical positionality refers to goods whose value is inversely related to the degree to which others have access to them. Horizontal positionality refers to goods whose value depends on the accessibility of other goods (van den Hoven et al. 2012). Different forms of positionality can be distinguished in the case of information goods (Zinnbauer 2018). These can either be the result of strategies to create artificial scarcities to obtain a positional advantage or the result of practical constraints that hamper the fluent distribution of information goods. Multiple forces that shape data frictions can be distinguished (Bates 2018): (1) data-sharing infrastructures, (2) socio-cultural factors, and (3) regulatory factors. Such frictions are claimed to have a “politics” because they shape the interactions between parties involved in data handling and exchanges (Bates 2018). Data-sharing infrastructures can introduce friction because of the complexity of the data representations needed to capture biological data, the lack of generally accepted data standards and ontologies, the anonymization and encryption methods required to guarantee genomic privacy, a variety of technical constraints such as bandwidth or computational power when dealing with big amounts of sequence data, and so on. A lack of data standards and data interoperability, for instance, was put forward as a challenge to open science in the Organisation for Economic Co-operation and Development’s (OECD’s) policy response to COVID-19 (OECD 2020). Data friction can also arise because of a lack of time or skills to cleanse, prepare, and submit the data, or a lack of time for scientists to document their experiments and annotate the data. Socio-cultural factors can legitimize data frictions, for instance, by guaranteeing the data privacy of research subjects or patients or avoiding a misinterpretation of the data. Socio-cultural data frictions also arise in highly competitive environments where data are retained in an explicit attempt to retain a competitive advantage or are shielded from scrutinization by other researchers (Bates 2018). Synthetic biology, in this regard, combines an open-source ethos and an intertwinement with commercialization activities. Often, the core members of the synthetic biology community are systematically in close proximity to commercial activities (Raimbault et al. 2016). A culture of data sharing is deeply interwoven with this scientific field, while, on the other hand, information asymmetries are implied by the competitive publication and innovation landscape.

Data positionality provides a lens through which to interpret the effects of data frictions in both highly competitive and highly collaborative research and development triggered by the COVID-19 pandemic. Various forms of data positionality can be distinguished. Temporal positionality refers to data goods in which the time component drives the differences in data accessibility. For instance, being able to run a speed-trading algorithm on servers next to the stock market can provide a few milliseconds of earlier access that an algorithm needs to outperform competitors. An analogous situation holds true for biological data. For instance, access to the SARS-CoV-2 genomic sequence and to epidemiological information proved to be crucial in effective policy and technological responses to the pandemic, for instance, in terms of the ability to rapidly develop diagnostic tests (Peeri et al. 2020). Hence data-release policies are important in shaping open environments where optimal use is made of research data. Building on the experiences from previous viral outbreaks, data-sharing platforms such as GISAID (GISAID 2020) and data-sharing guidelines (RDA COVID-19 Working Group 2020) were put in place. Geographical positionality refers to the competitive advantage that results from proximity to the location where the data are generated. It is easier to derive value from a dataset if one has direct insight into how the data were generated and processed, and if one has personal connections with the researchers who were involved in the process and one can tap into their tacit knowledge. The tight link between the data and the human population from which they were derived also ties data to a specific region. Biobanks, for instance, constitute a key resource in the fight against pandemics (Vaught 2020) and these repositories have physical locations. Population genomics and electronic health records are often bound to local populations and national initiatives. National borders (as a proxy for national regulations and political assessments) can result in data frictions and the related positional effects. For instance, for human genomic material and derived information, genomic sovereignty was proposed to ensure “a nation’s ability to capture the value of its investments in the field of genomic medicine” (Hardy et al. 2008). Getting access to data can require personal connections to scientists in heavily affected regions, for instance, when pursuing association studies (Olena 2020). Association studies—relating the genetic profile of individuals to their disease outcome—will be a key tool in answering the question of why SARS-CoV-2 hits patients with varying severity. Data-sharing initiatives, such as the COVID-19 Host Genetics Initiative, can help in reducing geographical positional effects in this case (The COVID-19 Host Genetics Initiative 2020). It is important to note that technical abilities and know-how are as crucial as the datasets that fuel the innovation process—and these assets can be equally positional. These innovation capabilities are geographically unequally distributed. For instance, investments in synthetic biology ventures in general in the second quarter of 2019 amounted to 1.2 billion USD, only 12% of which occurred outside of the USA (SynBioBeta 2019).

Owning positionality refers to situations in which ownership of the information good results in positionality. Next to keeping data in databases with restricted access, layered access and delays in data release are inherent to the biomedical field. This creates sub-domains that span a range from closed and proprietary, to knowledge commons that are managed by a community, up to databases that are geared toward open access. The transparency of proprietary data services (and of the quality of data in general) came under close scrutiny in the context of COVID-19 research, with the retraction of two high-profile papers (Piller and Travis 2020). One of the publications impacted trials with the drug hydroxychloroquine and the other led to increased demand for the drug ivermectin. In the case of monopolized positionality, single parties own the key data assets in a certain market. Such a situation also relates to new frontier positionality, referring to situations where parties gain a competitive advantage by entering into novel data fields. Synthetic biology is a field par excellence where dedicated technologies are developed and related information is gathered around cutting-edge fields of research. For instance, the extremely rapid development of mRNA- and DNA-based vaccine candidates for SARS-CoV-2 hinged on disruptive technologies that had already been explored in the context of other diseases.

Horizontal positionality occurs when the value of an information good depends on access to other information goods (van den Hoven et al. 2012). This is very often the case for biological data since the combined analysis of multiple biological datasets is often needed in order to derive value from them. Mining a population’s genomes in combination with medical records can provide powerful insights into disease trajectories and tailored treatments for specific sectors of the population (Boeck Jensen et al. 2014). For instance, the UK Biobank is going to add COVID-19 health-related data to its records, providing an integrated dataset for researchers to study the relationship between a person’s genetic makeup and disease susceptibility (UK Biobank 2020). Heterogeneous datasets often end up in separate, specialized data repositories with their own specific data-release schemes, access policies, and technical accessibility, which results in frictions when connecting the data. Big-tech positionality (Zinnbauer 2018) refers to the positional advantage that big companies and institutes can have when integrating data because of their access to significant amounts of proprietary data and to their data-analysis capabilities.

## Organizing responsible innovation in view of data positionality

To organize responsible innovation, one needs to be “response-able”—to be able to respond to the novel opportunities and risks that emerge. Previous viral outbreaks with pandemic potential proved that the level of data sharing significantly impacts this ability to respond. Responsiveness was highlighted as one of the dimensions of RRI (Stilgoe et al. 2013). It requires the ability to swiftly steer an innovation process if deemed appropriate. The COVID-19 pandemic demanded a quick innovation response to deliver therapeutics, vaccines, and diagnostics. This speed of response must go hand in hand with mechanisms to ensure ethical correctness and societal desirability. Aspects such as patient safety and data privacy, as well as dialogues around the desirability of novel therapeutic and preventative technologies, need to be interwoven with the entire innovation process. On the positive side, data positionality can foster competition in a Schumpeterian scheme, and thereby it can become instrumental in pursuing innovations that match societal preferences. However, leaving everything up to a market dynamic can also result in unequal data distribution and an unequal capability in terms of innovating and steering innovations. The COVID-19 pandemic led to a strong international push to mitigate temporal and geographical data positionality by strengthening rapid data-release and data-sharing mechanisms across national and institutional boundaries. For instance, the initial sharing of viral sequences via existing data-sharing mechanisms, such as GISAID (2020) and GenBank (GenBank SARS-CoV-2 2020), provided the necessary information for academic labs and companies to synthesize parts of the viral hereditary material, thereby vastly speeding up innovation processes globally. Synthetic biology approaches to COVID-19 aim at dramatically shortening the development of vaccines, therapeutics, and diagnostics. The hope is that the sheer diversity of innovations (Thanh Le et al. 2020) will provide room for responsiveness in terms of shaping the overall solution space.

Public debates on new technologies also often revolve around anticipation, reflexivity, and inclusion. Next to responsiveness, these perspectives together reflect societal concern and interest in technological innovation and can be used as dimensions in a responsible innovation approach (Owen et al. 2012; Stilgoe et al. 2013). Along these lines, the anticipate, reflect, engage, act (AREA) framework from the 2014 Rome Declaration (European Commission 2014) was recently proposed as a step toward RRI in COVID-19-related data research (Leslie 2020; Braun et al. 2020). These dimensions have been considered in the context of synthetic biology (Macnaghten et al. 2016) and healthcare (Silva et al. 2018). Next to the aforementioned dimensions, value domains specific to RRI in healthcare were proposed. These include, for instance, health equity, the level of care, frugality (if more can be achieved with fewer means), and the values that are embedded in the business model of the innovators (Silva et al. 2018). Anticipation is about considering possible outcomes of new technologies. Reflexivity means taking a step back and considering the innovation activities from a broader perspective. Data positionality should be included in assessments about anticipation and reflexivity, given the potential contribution to unintended consequences, and the effect on the desirability of the possible futures that the innovations will contribute to. Synthetic biology solutions, such as universal vaccines, for instance, have a “plug and play” character, and their effectiveness is thus tied to data availability. Geographical and temporal positionality will therefore be at play when such solutions will require tailoring to new variants of viruses. Owning positionality and big-tech positionality will relate to the question of how the landscape of providers and beneficiaries should be organized. Inclusion is a central theme in RRI approaches (Burget et al. 2017; Bogner and Torgersen 2018). Many stakeholders do not often have a say in the development of new technologies, although they need to bear the consequences later on. Healthcare innovations, for instance, have an impact on many of us; nevertheless, these innovations often take place in the confined labs of academic and corporate research institutes. For this reason, broad stakeholder involvement in the early innovation steps is often targeted in responsible innovation approaches (Stilgoe et al. 2013; Owen et al. 2013). This is in contrast to risk-assessment methods where the technical experts are the main driving forces and where assessments of the novel technologies are mainly done when approaching the market. The various types of data positionality can negatively impact this ability to include stakeholders. Competitive advantage is central to the very notion of positionality. Positionality therefore implies a topology in which the innovation step is shielded from external parties. Traditional models of drug innovation took place in the well-shielded environment of pharmaceutical companies, building on public data, but also deriving competitive advantage from proprietary data. These models are increasingly opened up in public–private partnerships that allow for deeper involvement of the public and the many stakeholders (biobanks, researchers, public healthcare funders, etc.), and in open innovation models. Such openness in the early innovation step implies a reduction in data friction.

The high sense of urgency related to the COVID-19 pandemic highlighted the importance of data access, data quality, and capabilities to put data to use. The high speed at which data-driven innovation occurs stresses existing processes. Central governance provides one route to shape data-driven innovation by modulating the effects of data positionality. Governance instills reciprocity among stakeholders via rules that constrain the options each individual rational party can select. Rules and regulations partially constrain the freedom of the individual players; nevertheless, they can be to their overall benefit by avoiding resource-wasting situations (Frank 2011). Data-privacy rules, for instance, constrain the space of innovations, thereby avoiding innovations that do not adhere to the imposed privacy values. Data-release policies, such as the open-science movement (Levin et al. 2016; Burgelman et al. 2019), can result in a broadening of the innovation space. Intellectual property arrangements provide an institutionalized way to create positional assets. Patenting of naturally occurring genetic sequences is no longer allowed by the United States Patent and Trademark Office, but it is still allowed in Europe (Cole 2015). Whether patenting genomic sequences has a positive or negative impact on healthcare innovation remains under debate (Liddicoat et al. 2019). Central governance may also be needed to contain data positionality to the sphere of research and innovation. For instance, the race for prime access to personal biological data should not negatively impact a person’s level of healthcare, career opportunities, or family. Avoiding “informational injustice” (Manders-Huits and van den Hoven 2008) requires rules and regulations that install data frictions at the boundaries of societal spheres.

In light of the ongoing pandemic, innovators were confronted with both aggressive timelines and high technological uncertainty. This situation triggered a vast increase in collaborations across highly diverse parties, leading to “a culture of collaboration across government, industry and academia” (Ledford 2020). This situation hints at future routes for responsible innovation. Fostering self-regulation and self-governance in innovation communities can be a venue for guiding innovation toward societally preferred goals. Research and innovation communities centered around synthetic biology can provide an entry point. A growing body of literature analyzes biological data as a common-pool resource—more precisely, as information goods in a knowledge common (Strandburg et al. 2017). This common’s perspective is helpful in clarifying the complex rules and the self-regulating properties of biological data-driven research and innovation communities. It also provides a framework to develop communities that mitigate negative positional effects and foster positive effects. The exclusion of certain stakeholders in an information common has been brought forward as an important element in the shaping of power asymmetries (Prainsack 2019). In pharmaceutical commons, positionality can be a driving force for gathering stakeholders around an innovation topic. Intellectual property and privately owned assets, when not used to fence off competitors, can function as a magnet to attract parties into collaboration (Lezaun and Montgomery 2015). For an individual party, it constitutes a “ticket of admission” to pharmaceutical product-development partnerships. Carefully managing access to data can also be a strategy in mitigating cybersecurity risks related to pathogen databases (Vinatzer et al. 2019) and confining the data to an innovation community adhering to strong research ethics. In these settings, certain types of positionality can be transformed into a creative source rather than a wasteful situation.

## Conclusion

The pace of synthetic biology innovations in response to the COVID-19 pandemic is unprecedented. This dynamic is driven by significant progress in synthetic biology as well as by improvements in data sharing. The potential societal impact of synthetic biology innovations calls for ways in which to foster beneficial outcomes that resonate with societal values while avoiding potential negative effects. Responsible innovation has been proposed as a framework to achieve this goal. When applied to synthetic biology innovations related to the COVID-19 vaccine, therapeutic, and diagnostic developments, it is clear that the role of data is pivotal. In this paper, biological data used in synthetic biology are analyzed as positional information goods. Positionality refers to the observation that the value of some pieces of information is related to their exclusivity. Various flavors of positionality can be identified relating to different types of data-access hurdles. Data positionality is Janus-faced—it can hamper responsible innovation but can also be a stimulating force. Measures to shape the data topology in terms of positionality are therefore an important instrument in steering synthetic biology innovations toward societally preferred goals. Central governance and self-governance in commons-like settings provide venues to mitigate the negative effects of data positionality.
